# Simultaneous Acquisition of Magnetic Resonance Elastography (MRE) and Diffusion Tensor Imaging (DTI) Optimized for Human Brain

**DOI:** 10.1002/mrm.70396

**Published:** 2026-05-03

**Authors:** Shujun Lin, Bradley P. Sutton, Richard L. Magin, Aaron T. Anderson, Dieter Klatt

**Affiliations:** ^1^ Richard and Loan Hill Department of Biomedical Engineering University of Illinois at Chicago Chicago Illinois USA; ^2^ Biomedical Imaging Center Beckman Institute, University of Illinois Urbana Champaign Urbana Illinois USA

**Keywords:** brain microstructure, diffusion tensor imaging, DTI‐MRE, intravoxel phase dispersion, magnetic resonance elastography, shear stiffness

## Abstract

**Purpose:**

Increase clinical acceptance of MRE and DTI by shortening the scan time by half and providing diffusive and mechanical property maps that are inherently co‐registered. This proof‐of‐concept study evaluates the simultaneous DTI‐MRE acquisition in the human brain and compares its results to conventional DTI and MRE measurements.

**Methods:**

We optimized experimental parameters for in vivo human brain DTI‐MRE acquisitions based on signal‐to‐noise ratio, encoding efficiency, and intravoxel phase dispersion mitigation. Five healthy subjects underwent scanning on a 3 T Siemens Prisma scanner, using two optimized parameter sets. Diffusive (mean diffusivity, fractional anisotropy) and mechanical (shear stiffness) property maps were computed and compared with conventional methods using Pearson's correlation analysis.

**Results:**

DTI‐MRE correlated with conventional DTI and MRE methods across all subjects. Mean diffusivity and fractional anisotropy maps showed excellent fidelity between methods (Pearson's coefficients of global mean values > 0.86, Pearson's coefficients of voxel‐wise values > 0.7). Spatially averaged shear stiffness values were slightly lower in DTI‐MRE.

**Conclusion:**

This proof‐of‐concept study confirms the feasibility of simultaneous DTI‐MRE acquisition in the human brain, offering an efficient method for obtaining both diffusive and mechanical property maps. Further refinements in encoding strategies and inversion algorithms, such as the incorporation of a multi‐frequency actuation approach in the DTI‐MRE framework, could enhance accuracy, enabling broader clinical applications in neurology, neurosurgery, and brain injury assessment.

## 1 Introduction

Magnetic resonance elastography (MRE) and diffusion tensor imaging (DTI) are noninvasive MRI techniques that characterize the mechanical and diffusive properties of biological tissue under various pathological conditions. MRE applies three motion‐encoding gradients to acquire snapshots of the continuous harmonic vibration in 3D space, from which shear stiffness maps can be reconstructed using inversion techniques. DTI encodes the random motion of water molecules and their restriction to movement in tissue by applying multiple encoding gradients. Researchers have applied MRE and DTI individually for assessing the diagnostic potential of the methods for various pathological conditions. For example, Pavuluri et al. reported a significantly lower brain stiffness of Alzheimer disease (AD) patients compared to a cognitive normal control group in an MRE study [1]. In another MRE study, Duhon et al. assessed the tumor stiffness to predict surgical outcomes in vestibular schwannoma and meningioma [2]. Recent studies used DTI metrics as in vivo markers for detecting protein‐related brain atrophy in AD [3]. In addition, DTI can also provide fiber tract information of spinal cord and brain for chronic traumatic spinal cord injury [4]. However, for each of these clinical conditions, neither diffusivity nor stiffness maps alone can provide a full picture of neurological diseases.

Besides just acquiring DTI and MRE in the same patient, researchers have combined the information from DTI and MRE for better comprehension of the microstructural composition in regions of the brain. Romano et al. developed the waveguide elastography (WGE) approach, which uses DTI to evaluate the fiber pathways of the corticospinal tracts (CSTs) and then reconstructs anisotropic shear stiffness maps using the directionality of CSTs as one of the inputs for anisotropic wave field inversion. In a patient study, this combined DTI and MRE approach yielded mechano‐structural metrics that are sensitive to amyotrophic lateral sclerosis (ALS) [5]. Combination of DTI and MRE also shows the potential to assess the tumor cell invasion and radiotherapy responses in experimental glioma [6]. A recent study also explored the combined use of DTI and MRE in the preoperative assessment of meningioma structure and consistency [7].

The clinical studies mentioned above acquired the DTI and MRE data in separate, successive acquisitions, expanding the clinical study time and requiring registration for comparison. However, the structure of the two acquisitions shares many features including the use of multiple direction motion encoding gradients, a spin echo acquisition, and similar EPI‐based acquisition readouts. The two sequences make their different measurements of tissue properties through the different extent of gradient encoding and the use of synchronized mechanical actuation. The feasibility of rapid, simultaneous acquisition of DTI and MRE data has previously been demonstrated in a preclinical in vivo study on mouse brain [8]. The benefits of simultaneous DTI and MRE acquisitions, which is named DTI‐MRE, include reduced scan time (factor of ˜2) and the receipt of immediately co‐registered diffusive and mechanical property maps. However, this approach can result in intensity variations in the MRI magnitude images, where wave patterns may be imposed by subvoxel deformations. This effect is referred to as intravoxel phase dispersion (IVPD) [9] and can potentially interfere with the assessment of diffusion data based on MRI magnitude image analysis. This interference can be minimized by selecting experimental parameters that achieve the encoding needs but minimize the interaction effects so that accurate simultaneous DTI and MRE acquisitions are obtained.

In the present proof‐of‐concept study, we test the mixing of DTI and MRE by performing a careful matched comparison with standard sequence to keep the mixing of DTI and MRE clear. To this end, we used simulations to identify sets of experimental parameters that minimize IVPD, then employed these parameters to obtain concurrent in vivo human brain DTI‐MRE on five volunteers. Finally, we assess the influence of different IVPD levels on the reproducibility of diffusion and mechanical property maps, comparing them with maps acquired separately using the conventional methods.

## 2 Theory

### 2.1 Gradient Encoding Efficiency in DTI‐MRE

**FIGURE 1:**
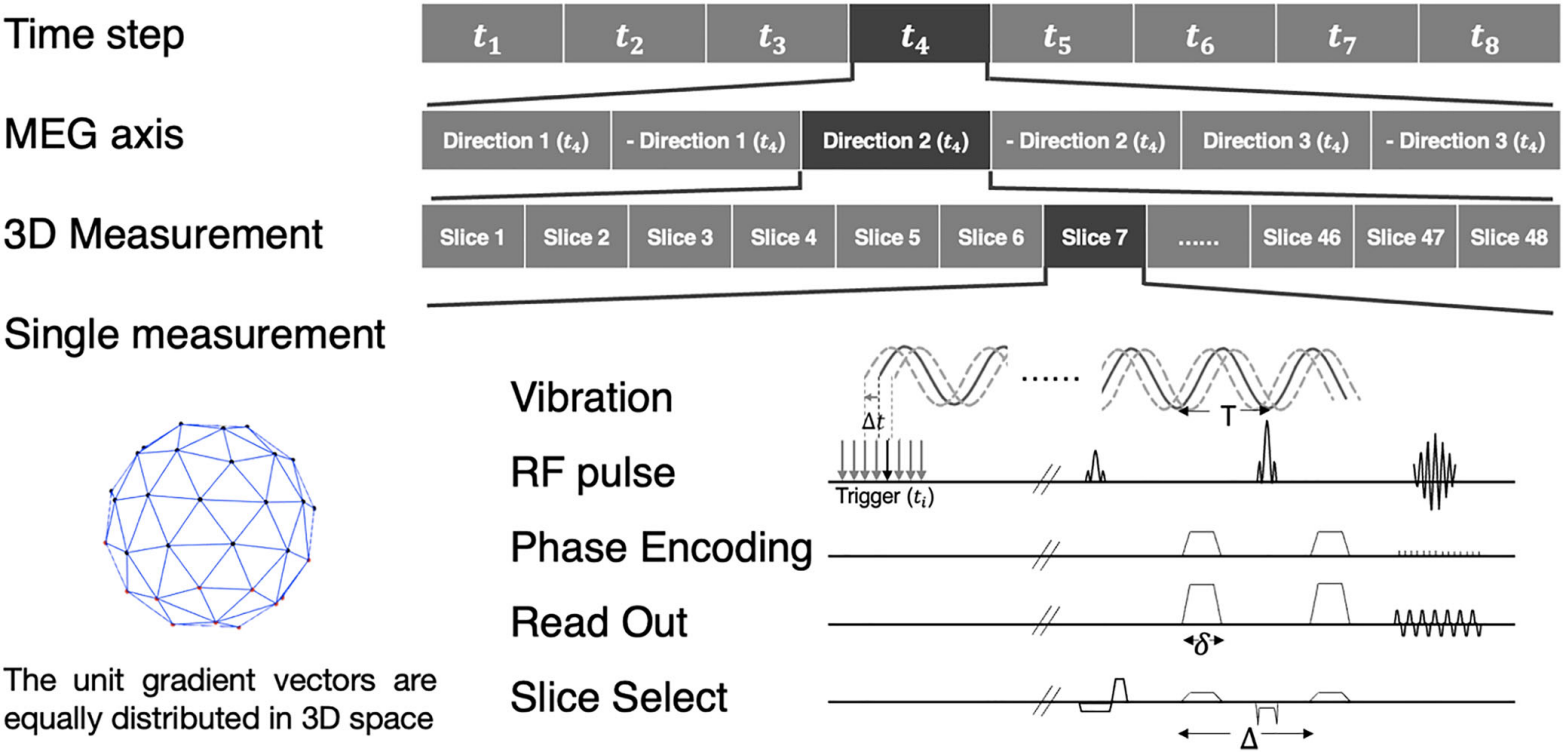
Sequence regime. A single‐shot echo‐planar‐imaging based MRE sequence was modified to fulfill the DTI‐MRE encoding regime. Eight phase offsets are acquired, where each phase offset consists of three encoding directions with positive and negative polarities. The trigger signal for vibration was sent after acquiring the b0 image (b0 measurement not shown in figure) for all slices.

Concurrent diffusion and elastography acquisitions in tissue are feasible, as reported in previous studies [8, 10], in the pulsed gradient spin echo (PGSE) framework, by application of a gradient lobe on each side of the refocusing pulse in a spin‐echo based MRI sequence. For efficient MRE acquisitions, the vibration phase at the end of the first gradient lobe must be the same as that at the start of the second gradient lobe. Therefore, the condition of simultaneous encoding in DTI‐MRE requires the separation time between the gradient lobes to correspond to an integer multiple of the vibration period, giving a diffusion mixing time ∆:

(1) ∆=δ+nT,

where *T* is the vibration period, *δ* is the gradient lobe duration, and *n* is an integer. Figure 1 illustrates the described scenario. This way, the vibratory whole voxel movement and the diffusive motion of water molecules within the voxel can be encoded into the MR signal phase and magnitude, respectively.

The diffusion signal encoding of the gradient is characterized by the well‐established b factor,

(2) $$S\left(b\right)=S_{0}e^{-\mathrm{bD}}$$

(3) $$b=\gamma^{2}\int_{0}^{\mathrm{TE}}{\left[\int_{0}^{t}G\left(t^{\prime }\right)dt^{\prime }\right]}^{2}\mathrm{dt},$$

where S is the diffusion‐attenuated magnitude of the MRI signal, $S_{0}$ represents the signal magnitude without diffusion encoding, D is the apparent diffusion coefficient in the gradient direction, γ is the gyromagnetic ratio, TE corresponds to the echo time of the spin‐echo sequence and G(t) describes the gradient waveform as a function of time.

The mechanical phase Φ that voxels will accumulate during application of the gradients when experiencing harmonic vibrations with period *T* in parallel to the gradient direction, peak displacement $u_{0}$, and with initial mechanical phase $\varphi_{0}$ at the onset of the gradient can be calculated by [11]:

(4) $$\Phi =\gamma \int_{0}^{\mathrm{TE}}u_{0}\sin\left(\frac{2\pi }{T}t+\varphi_{0}\right)\cdot G\left(t\right)\;\mathrm{dt}.$$

Integral Equation (4) results in a harmonic function of $\varphi_{0}$ with amplitude $\Phi_{0}$. After solving Equation (4) for a specific gradient waveform the vibration encoding efficiency of the gradient (ξ), can be denoted as:

(5) $$\xi =\frac{\Phi_{0}}{u_{0}}.$$

This amplitude ratio quantifies the accumulated MRI signal phase per meter displacement amplitude.

### 2.2 Linking Gradient Directions to MRE Phase Offsets in DTI‐MRE

In DTI, the goal is to sample a number of diffusion encoding directions, but in MRE the goal is to get three nearly orthogonal encoding directions sampled at multiple time points along a period *T*. The linking of gradient directions to MRE phase offsets in DTI‐MRE has been introduced in detail in (Yin et al. 2017). In short, the number of gradient directions for DTI must be an integer multiple of three so that three directions can be assigned to one MRE phase offset. To ensure accuracy of DTI quantification, all gradient directions are equally distributed on a 3D hemisphere, which can be achieved by minimizing the electrostatic energy of charged electrical particles on a unit hemi‐sphere [12]. Finally, the full 3D MRE displacement can be captured by sustaining the highest degree of linear independence for the three gradient directions at each MRE phase offset. This condition is realized by minimizing Equation (6),

(6) $$\min\sum_{q=1}^{N_{\mathrm{po}}}\left(\left|e_{1}^{q}\cdot e_{2}^{q}\right|+\left|e_{1}^{q}\cdot e_{3}^{q}\right|+\left|e_{2}^{q}\cdot e_{3}^{q}\right|\right),$$

where $N_{\mathrm{po}}$ is the number of MRE phase offsets, and $e_{i}^{q}$ corresponds to a unit vector of one of the three gradient directions assigned to the qth MRE phase offset.

### 2.3 Balancing Gradient Encoding Efficiencies and Vibration Induced Signal Loss

Simultaneous acquisition of DTI and MRE induces magnitude signal loss due to IVPD, which results in an overestimation of the mean diffusivity (MD). Pulse sequences that minimize the signal loss due to the IVPD effect can be determined by varying the experimental parameters for DTI and MRE.

In this proof‐of‐concept study we adopted the signal efficiency model derived by Yin et al. [8] that quantifies IVPD‐induced signal loss by means of the ratio, *R*, of the magnitude signal considering a shear wave propagating along the *x*‐direction compared to the magnitude signal in the static case. In Equation (7), *x*_c_, Δ*x, λ*, *u*_0_, and *ξ* represent the voxel center, voxel size along *x*, the shear wavelength, shear wave amplitude, and the vibration encoding efficiency, respectively.

(7) $$R\left(x_{c}\right)=\frac{1}{\Delta x}\left|\int_{-\Delta x/2}^{\Delta x/2}\exp\left(i\left[\xi \cdot u_{0}\cdot \sin\left(\frac{2\pi }{\lambda }x+x_{c}\right)\right]\right)\mathrm{dx}\right|$$

For a gradient waveform of two single rectangular lobes separated by the diffusion time Δ, the vibration encoding efficiency *ξ* and the diffusion encoding efficiency *b* are described by Equations (8) and (9), respectively,

(8) $$\xi =\frac{\gamma G_{0}\left(1-\cos\left(\pi T_{g}f_{v}\right)\right)}{\pi f_{v}}$$

(9) $$b={\left(\gamma G_{0}\delta \right)}^{2}\left(∆-\frac{\delta }{3}\right),$$

where *G*_0_ represents the gradient amplitude, *T*_g_ = 2*δ* is the combined duration of both gradient lobes and *f*_v_ corresponds to the vibration frequency. As outlined in the methods section, Equations ((7), (8), (9)) are employed to optimize the experimental parameters in DTI‐MRE based on the best compromise between signal loss and gradient encoding efficiencies.

## 3 Methods

### 3.1 Optimization of Experimental Parameters

In the general case, experimental parameter ranges suitable for DTI‐MRE are determined using Equations ((7), (8), (9)) and observing the following conditions [13]: (1) short TE for obtaining high signal‐to‐noise ratio (SNR); (2) vibration frequency within a reasonable range to impose at least one half wavelength across the region of interest; (3) sufficient motion encoding efficiency for encoding displacement to the phase of the MRI signal; (4) the signal loss due to IVPD that can interfere with magnitude images needs to be controlled within an acceptable error margin; (5) the timing condition (Equation (1)) for DTI‐MRE must be fulfilled.

For the optimization of the experimental parameter range in our in vivo human brain DTI‐MRE study, we assumed a vibration amplitude of 25 μm and an isotropic voxel size of (3 mm)^3^, and we stipulate the below specific conditions: (1) the total duration of the encoding process, that is, Δ + *δ*, not exceeding 60 ms; (2) 1 Hz vibration frequency increments within range of 20 –70 Hz; (3) motion encoding efficiency larger than 1.5 × 10^5^ rad/m; (4) the signal loss due to IVPD to be less than 25%; (5) the timing condition (Equation 1) for DTI‐MRE must be fulfilled; (6) *b*‐value within range of 980–1020 s/mm^2^, which is typically used in in vivo human brain DTI [14, 15, 16, 17]; (7) shear wave length in Equation (7) calculated by dividing the assumed average shear wave speed in human brain of 1.7 m/s [18] by the vibration frequency.

We performed an exhaustive search of parameter ranges for the vibration frequency *f*_v_, the gradient amplitude *G*_0_, the number of vibration cycles in gap (*n* in Equation (1)) and the ratio of combined gradient lobe duration and vibration period, which can be expressed as *f*_v_ × *T*_g_. The raster size in our search was *f*_v_ = [20 Hz…70 Hz] in 1 Hz steps, *G*_0_ = [10 mT/m…80 mT/m] in 0.1 mT/m steps, *n* = [1 2 3] and *f*_v_ × *T*_g_ = [1/2 3/4 1 5/4 3/2]. Acceptable results are all parameter combinations in the search, which obey the seven restrictions specified above.

### 3.2 Subjects

Five healthy subjects in the age range 20–60 years were recruited and scanned on a 3 T Siemens Prisma scanner (Siemens Medical Solution, Erlangen, Germany) and a 64‐channel head coil. Written consent forms that were approved by Institutional Review Board (IRB), University of Illinois Chicago, were collected from all subjects.

### 3.3 Vibration Setup

The harmonic vibration was delivered to the subject through a pillow passive driver positioned at the back of the head, which was controlled by the pneumatic device of an MRE system (Resoundant, Rochester, MN) outside of the scanner room.

### 3.4 Data Acquisition

A single‐shot spin‐echo echo‐planar‐imaging (SS‐SE‐EPI) MRE sequence was modified to realize the simultaneous encoding of diffusion and vibration, as shown in Figure 1. There were 24 encoding directions with opposite polarities in DTI‐MRE that were grouped in eight groups; each group had three encoding directions with maximized linear independence and corresponded to one of the eight phase offsets in vibration encoding for MRE. Images without motion encoding were acquired for all slices before sending the trigger signal for vibration.

Experimentally we tested the performance of two optimized parameter sets that have different signal loss due to IVPD. Ideally, the parameter set with less signal loss and higher min{$R\left(x_{c}\right)$} would provide property maps with higher accuracy, while employing the set with relatively more signal loss and moderate min{$R\left(x_{c}\right)$} should provide property maps within acceptable, but wider error margins. The sequence used a TE = 80 ms, a matrix size of 80 × 80 and a voxel size of 3 mm isotropic and 8 phase offsets and 48 oblique axial slices, approximately aligned with anterior–posterior commissures were acquired. The 40 Hz (50 Hz) vibration experiment employed 6 (7) vibration cycles resulting in a TR = 150 ms (140 ms). Thus, all 48 axial slices of one time step and of one MEG direction could be obtained in 7200 ms (6720 ms) using continuous vibration. Further sequence parameters are listed in Table 1. For comparison, conventional DTI and MRE acquisitions were performed. The DTI acquisition used the same parameters as in DTI‐MRE but without vibration. The MRE acquisition used a gradient amplitude of 78 mT/m and the same vibration frequency as in the optimal parameter sets while the encoding gradient lobe duration was set to half of the vibration period. For both the MRE and the DTI‐MRE acquisitions, the repetition time (TR) was adjusted to allow continuing vibration during acquisition. The DTI acquisition used the same TR for consistency. At least 50 ms of vibration was included to allow the wave onset before motion encoding was applied. The scan time for individual DTI‐MRE, MRE, and DTI acquisitions was approximately 5 minutes and 40 seconds.

**TABLE 1:** The chosen optimized parameter sets with *b*‐values 982 ± 2 s/mm^2^ from simulation results.

	Frequency of vibration (Hz)	Ratio = *f*_v_/*f*_g_	Frequency of dMEG (Hz)	Encoding efficiency (10^5^ rad/m)	min {$R\left(x_{c}\;\right)$} (%)	Diffusion time (ms)	Gradient amplitude (mT/m)
PS1	40	1.25	32.0	1.6	87.6	40.6	43.8
PS2	50	1.25	40.0	1.8	75.7	32.5	62.8

### 3.5 Data Analysis

The magnitude images were used to extract diffusion information along all encoding directions, followed by calculating the eigenvalues and eigenvectors of the diffusion tensor. Thereafter, MD and FA maps were determined voxel‐wise.

**FIGURE 2:**
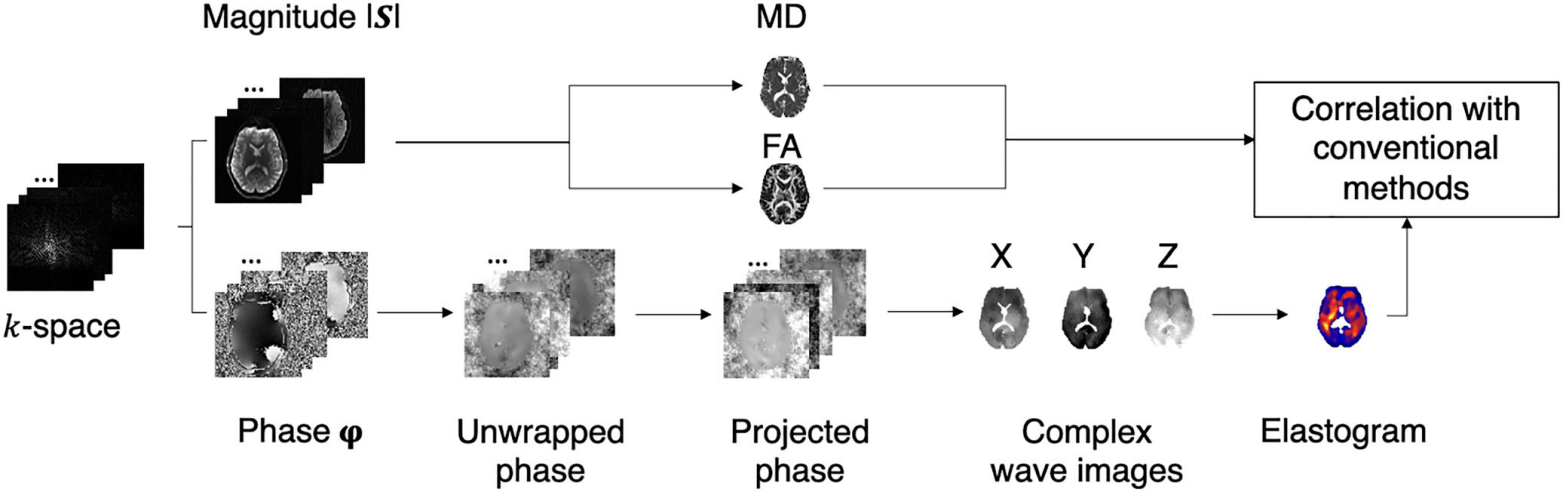
Pipeline of DTI‐MRE image processing.

The phase difference maps were obtained using the phase images acquired with opposite gradient polarities. Then a Flynn's unwrapping algorithm was applied to remove wrapped phases. In DTI‐MRE, the vibratory motion along three linear independent directions was encoded into the MR signal phase. In each voxel, the 3D phase vector was projected onto the principal directions (*x*/*y*/*z*) of the scanner. This projection step was not needed for analysis of the conventional MRE data, which were directly encoded along the principal directions. The complex wave images (CWIs) were extracted from the first harmonic after the Fourier transformation of phase images along the eight time‐steps, which was done for each directional component. A 3D Butterworth lowpass filter of 2nd order with low cutoff frequency of eight pixels was applied to each of the *x*‐, *y*‐, and *z*‐CWIs, for noise reduction. Subsequently, the spatial mean subtraction was applied to CWIs to remove an offset in the CWIs, which was typically a multiple of *π*. The curl operation was applied to the offset‐corrected CWIs for filtering out the contribution of the compressional wave [19]. Furthermore, the complex shear modulus was calculated by inverting the Helmholtz equation in a least‐square manner [20]. Figure 2 displays the processing pipeline in DTI‐MRE described above.

The image registration was done in FSL6.0.6 (FMRIL, University of Oxford) [21, 22]. For each subject, the affine matrix was generated by rigid‐body registration between *T*_2_‐weighted images and DTI‐MRE magnitude image, which were then applied to each property map. The spatial mean values of the central 16 brain slices excluding cerebral spinal fluid (CSF) in registered property maps were calculated.

### 3.6 Statistical Analysis

Correlation studies were performed on property maps of the 16 central slices between DTI‐MRE and conventional DTI/MRE acquisitions using Pearson's rank correlation. For each subject, the spatial correlation coefficients of the MD, FA, real and imaginary parts of CWIs in three principal directions were determined. For group analysis, correlation between DTI‐MRE and conventional methods using either parameter set on mean values of property maps was examined.

## 4 Results

As shown in Table 1, two optimized experimental parameter sets, that complied with the aforementioned conditions from simulation results, were chosen to be examined. Full table of simulation results can be found in Table S1. The *b*‐values for these experimental parameters were inclined to the lower boundary, since lower *b*‐values involve lower gradient amplitudes, which also tend to result in a reduction of the IVPD effect (see Equations (7), (8), (9)). The two parameter sets for the experimental study were chosen based on their representation of the upper and lower end of the IVPD signal loss range quantified by min{$R\left(x_{c}\right)$}, while including a vibration frequency value typically used in in vivo human brain MRE [23, 24, 25]. These parameter sets were denoted as parameter set 1 (PS1) with min{$R\left(x_{c}\right)$} = 87.6%, and parameter set 2 (PS2) with min{$R\left(x_{c}\right)$} = 75.7%.

**FIGURE 3:**
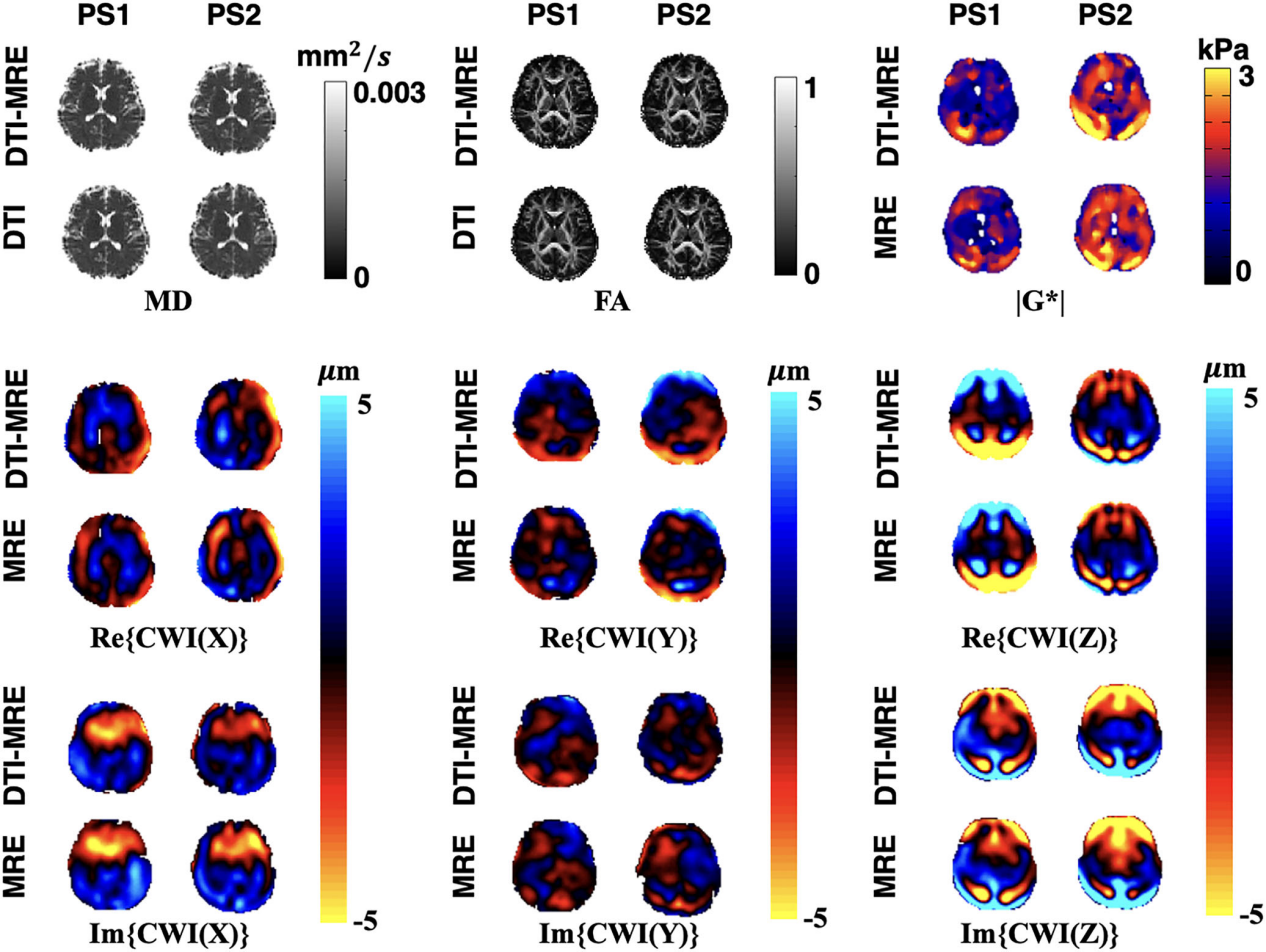
Diffusive and mechanical property maps (top row), real (middle row) and imaginary (bottom row) parts of displacement maps along the three spatial axes obtained using the two experimental parameter sets (PS1 and PS2) in a central axial slice of one volunteer. For comparison, the property maps processed from acquired data using conventional methods are also displayed.

Figure 3 illustrates for one subject the diffusive property maps, mechanical property maps, and real and imaginary parts of the displacement maps along the three spatial dimensions. For each subject, the spatial mean values over the central 16 brain slices, excluding CSF, of MD, FA, and |G*| maps using both parameter sets were estimated and are compared for DTI‐MRE and conventional methods in Table 2.

**TABLE 2:** Spatial mean values of MD, FA, and |G*| averaged over 16 central brain slices excluding CSF. The values are listed for each subject (A, B, C, D, and E) using DTI‐MRE or conventional DTI/MRE measurements.

	MD (μm^2^/ms)		FA		|G*| (kPa)	
	DTIMRE	DTI	DTIMRE	DTI	DTIMRE	MRE
A. PS1	0.96	0.96	0.25	0.25	1.72	1.81
A. PS2	0.95	0.95	0.25	0.25	2.33	2.36
B. PS1	0.91	0.91	0.25	0.25	1.72	1.80
B. PS2	0.91	0.90	0.25	0.25	2.36	2.57
C. PS1	0.87	0.86	0.26	0.27	1.47	1.78
C. PS2	0.84	0.84	0.26	0.27	2.07	2.51
D. PS1	0.84	0.84	0.27	0.27	1.56	1.90
D. PS2	0.83	0.83	0.27	0.27	2.35	2.50
E. PS1	0.82	0.82	0.27	0.27	1.46	1.72
E. PS2	0.83	0.82	0.27	0.27	2.42	2.57

**FIGURE 4:**
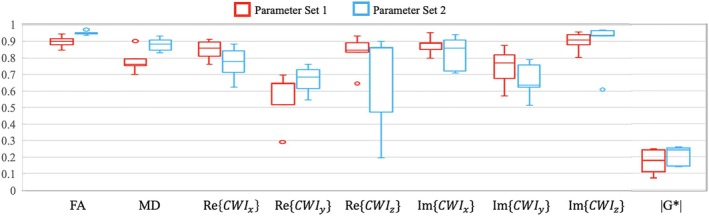
Spatial Pearson's correlation coefficients of DTI‐MRE and conventional DTI/MRE in each subject's 16 central brain slices excluding CSF for MD, FA, real and imaginary parts of complex wave images (CWI_*X*_, CWI_*Y*_, CWI_*Z*_), and absolute shear stiffness (|G*|).

The inter‐individual mean of central 16 brain slices, excluding CSF, on MD, FA and |G*| maps from PS1/PS2 using DTI‐MRE are (0.88 ± 0.06/0.87 ± 0.08 [μm^2^/ms]), (0.26 ± 0.05/0.26 ± 0.05) and (1.59 ± 0.11/2.31 ± 0.12 [kPa]), respectively. The percentage of difference of the interindividual means on MD, FA and |G*| maps between DTI‐MRE and conventional methods using the two parameter sets (PS1/PS2) are (0.1% ± 0.8% / 0.4% ± 0.7%), (−0.1% ± 1.9%/−0.4% ± 0.9%) and (−12% ± 5.8%/−8% ± 5.4%), respectively. Furthermore, Figure 4 depicts the boxplots of the spatial correlation coefficients on property maps of five subjects.

**FIGURE 5:**
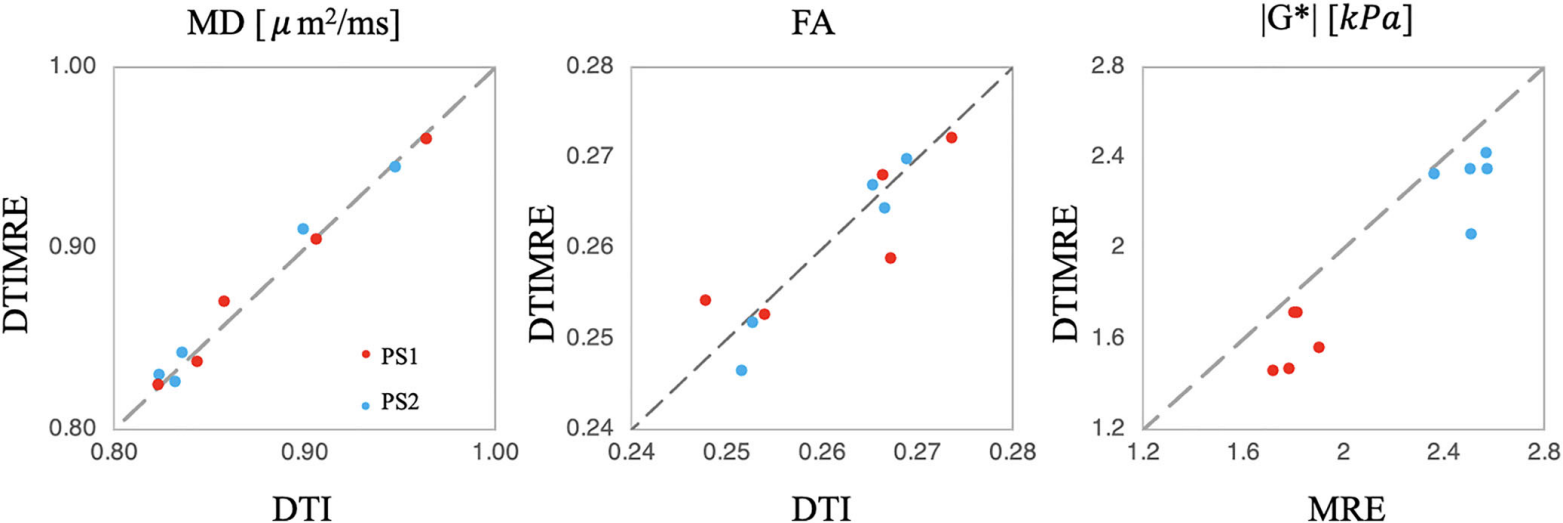
Correlation of mean values averaged over 16 central brain slices excluding CSF using DTI‐MRE and conventional DTI/MRE measurements for MD, FA, and |G*| maps. The dashed gray line represents the identity line.

The global averaged values on the central 16 brain slices of property maps using either PS1 or PS2 parameter sets are shown in Figure 5. The correlation coefficients of averaged values of MD, FA and shear stiffness for PS1/PS2 sets are 0.99/0.99, 0.86/0.98, and 0.33/0.13, respectively.

## 5 Discussion

This proof‐of‐concept study demonstrates the feasibility of in vivo human brain DTI‐MRE, which was implemented on a 3.0 T clinical MRI scanner. We have examined PS1 and PS2 and compared their performance to the results obtained using conventional methods. Overall, property maps using DTI‐MRE and conventional methods were similar, independent of the experimental parameter set used (PS1 or PS2). Remarkably, the diffusion metrics showed an excellent reproducibility between DTI‐MRE and DTI. Regarding the mechanical properties, two observations were noticed: (i) the stiffness values using the PS2 were higher than when using PS1. This observation can be explained by the frequency dispersion behavior of the shear modulus |G*| [26]. Of note, the vibration frequency used with PS2 was 50 Hz as compared to 40 Hz using PS1; (ii) the mean absolute shear stiffness from DTI‐MRE is slightly smaller than using conventional MRE among all subjects. This observation is likely due to the lower SNR using DTI‐MRE, which can affect the MRE results [27].

Based on our experimental study we cannot recommend a preference for either of the two applied parameter sets. IVPD‐induced signal loss within our considered margins does not seem to play a role regarding the reproducibility of property maps in DTI‐MRE compared to conventional acquisitions. Therefore, we can assume that all optimized parameter sets yield satisfactory results. The experimental study highlights the excellent reproducibility of the diffusion property maps. The correlation of mean stiffness values derived from DTI‐MRE with values obtained using conventional methods was not significant. Stiffness reconstruction from the MR phase images is an ill‐posed problem and can result in errors around voids of standing wave patterns. Since the spatial characteristics of standing wave patterns in a continuum will change with the mechanical actuation frequency, we suggest the incorporation of a multi‐frequency approach into the DTI‐MRE framework for stiffness reconstruction improvement. Such an alternative implementation of DTI‐MRE could incorporate two actuation frequencies tied to 12 directions each with one *b*‐value. However, using a different *b*‐value with each actuation frequency is also a possibility and can account for multiple *b*‐shells. On another note, the small sample size used in our experimental study (five volunteers) is a limitation. While we report in the results section that the correlation coefficient of the mean stiffness values for the five volunteers using the PS2 is 0.13, this value would increase to 0.73 if the outlier volunteer C in Table 2 was excluded.

In the present proof of concept study, we tested the mixing of DTI and MRE. We performed a carefully matched comparison to keep the mixing of DTI and MRE clear. We used sequences based on SS‐SE‐EPI with a TE = 80 ms in all experiments and chose a 3 mm isotropic resolution, which does not meet the current standards for either individual MRE or DTI protocols. The relatively low resolution enabled us to finish all scans (MRE, DTI, MRE‐DTI) for one volunteer within reasonable scan time. Optimization of DTI‐MRE sequences towards higher resolution and for specific organ targets represents future work. We also note that the DTI‐MRE concept is not bound to a specific sequence type. It can be integrated into other sequences using advanced methods. For example, multiband sequences that meet the current standard for either DTI or MRE can be applied, and the weighting between them is not expected to change.

We performed all experiments using a TE = 80 ms, which was the shortest possible echo time for PS 1. This enabled us to compare the performance of PS 1 and PS 2 based on the mixing of DTI and MRE due to IVPD‐induced signal loss while keeping T2‐relaxation fixed. However, the shortest possible TE for PS 2 is approximately 69 ms. The minimum value should be used in future studies, when PS 2 is employed, which maximizes SNR.

It is a limitation of DTI‐MRE of the brain that the encoding concept may be susceptible to jitter effects. The DTI part relies on *b*‐values of ˜1000 s/mm^2^, which can be realized only by using single lobes separated by the refocusing RF pulse within the constraints of our experimental parameter ranges. While gradients split into two lobes as shown in Figure 1 have the same frequency selectivity as bipolar gradients for the mechanical excitation frequency, this does not hold for lower frequencies due to, for example, physiological noise and cardiac pulsation. The increased sensitivity to low frequency motions can result in jitter effects, which can negatively impact stiffness estimation [28, 29].

Our parameter optimization protocol considers the conditions for in vivo human brain DTI‐MRE. We note that the assumed experimental parameter ranges for the vibration amplitude and encoding efficiency in our simulation recipe described in the methods section may be on the upper end of brain MRE protocols. However, lower values for vibration amplitude and encoding efficiency and higher spatial resolution will play in favor of reducing IVPD artifacts in DTI‐MRE [30]. While our conditions in the simulation recipe can be followed in principle, we note that other applications use different standards for the *b*‐value (DTI) and involve other mechanical wave characteristics (MRE). For example, in DTI of the human kidney the used *b*‐values can be as low as 400 s/mm^2^ [31] and preclinical MRE protocols result in displacement amplitudes well below 5 μm [8]. Therefore, each application may require the use of different values for the *b*‐value, displacement amplitude, vibration frequency and voxel size in the parameter optimization pipeline.

In summary, concurrent DTI and MRE acquisitions are feasible and the resulting property maps are in good agreement with the ones obtained using conventional acquisitions. While the diffusive property maps are remarkably similar, we speculate that the integration of a multi‐frequency vibration approach will positively affect the reproducibility of mechanical property maps. DTI‐MRE has the potential to increase the clinical acceptance of diffusive and mechanical diagnostic parameters by providing multi‐parametric information in approximately half the acquisition time compared to performing DTI and MRE in separate, sequential scans.

## Funding

This work was supported by the National Institutes of Health (R21EB026238).

## Supporting information


**Table S1:** Simulation results prior to in vivo studies. The optimization process returned *b*‐values of 982 ± 2 s/mm^2^ for all optimized parameter sets. Two sets (*) and (**) were examined and displayed in Table 1.

## Figures and Tables

**FIGURE A.1:**
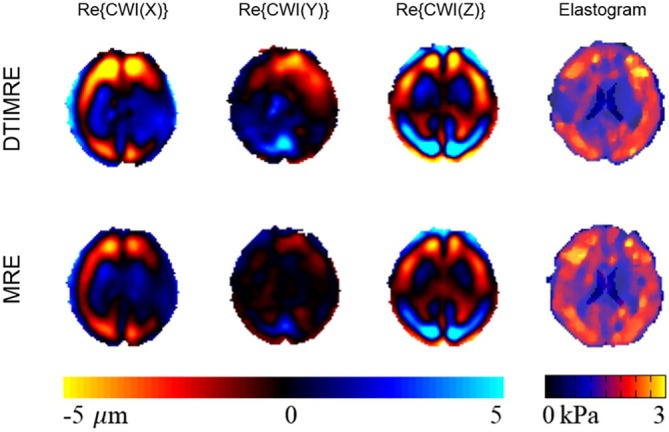
A direct comparison of displacement maps in three axes and absolute shear stiffness map between DTIMRE and conventional MRE with flow compensation.

**FIGURE A.2:**
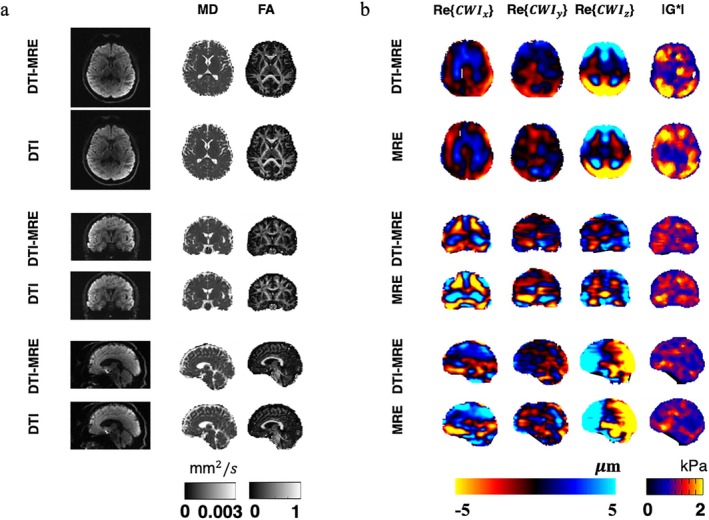
A direct comparison between DTIMRE and conventional methods in axial, coronal and sagittal views. Experimental parameter set PS1 (see Table 1) was used. (a) Magnitude images and diffusion property maps (MD and FA). (b) Displacement and absolute shear stiffness maps.

## Data Availability

The data that support the findings of this study are available from the corresponding author upon reasonable request.

## Appendix A

### Experimental Comparison of DTI‐MRE Using 0th Gradient‐Moment‐Nulled (GMN) and Flow‐Compensated MRE

Conventional MRE using flow compensated motion‐encoding gradients (MEG) and DTI‐MRE using MEG with only the 0th GMN were performed on in vivo human brain at the same mechanical frequency of 50 Hz. The echo time (TE), matrix size, voxel size, and number of slices were 78 ms, 100 × 100, 2.5 × 2.5 × 2.5 mm^3^ and 48, respectively. The gradient amplitudes for MRE and DTI‐MRE were 78 and 64 mT/m, respectively. Correlation analysis between DTI‐MRE and flow‐compensated MRE was carried out on wave images and property maps (|*G**|) using eight central brain slices.

Wave images filtered as described in the methods section, and |*G**| maps of a central axial slice acquired using DTI‐MRE and conventional MRE with flow compensation are displayed in Figure A.1. The Pearson's correlation coefficients for eight central brain slices in, *Re*{CWI(*X*)}, *Re*{CWI(*Y*)}, *Re*{CWI(*Z*)}, and |*G**| maps were 0.78, 0.51, 0.94, and 0.36, respectively. The wave structures in *X* and *Z* axes are nearly identical, leaving *Y* axis less ideal due to the relatively low displacement along that direction and possible flow contributions in the DTI‐MRE scan. However, it is evident by visual inspection that the reconstructed stiffness maps still exhibited similar features. Therefore, we proceeded with 0th GMN gradients in the DTI‐MRE protocol that enable *b*‐values in ranges typically used in human brain DTI unlike the flow‐compensated gradient waveforms.

### Comparison of DTI‐MRE and Conventional Methods in Three Orthogonal Planes

Figure A.2 illustrates a side‐by‐side comparison of the magnitude images and diffusion property maps (a) and of the complex wave images (real part) and stiffness maps between DTI‐MRE using parameter set PS1 (see Table 1) and conventional methods (b). The complex wave images are calculated by Fourier transform of the temporally resolved phase images. In DTI‐MRE, the 3D phase vector of each voxel was projected onto the principal directions (*x*/*y*/*z*) of the scanner as described in the methods section, while this projection step was not needed in conventional MRE. The complex wave images are used as input for the inverted Helmholtz equation as outlined in the methods section, which results in the shear stiffness maps.
