# Supplementary material for: Simultaneous Acquisition of Magnetic Resonance Elastography (MRE) and Diffusion Tensor Imaging (DTI) Optimized for Human Brain
Source: Magn Reson Med. 2026 May 3;96(3):1111–20. doi: 10.1002/mrm.70396 (PMC13327488; doi:10.1002/mrm.70396)
Supplement: Supplementary file 1 — Table S1: Simulation results prior to in vivo studies. The optimization process returned b‐values of 982 ± 2 s/mm2 for all optimized parameter sets. Two sets (*) and (**) were examined and displayed in Table 1. [file MRM-96-1111-s001.docx]

Table S1. Simulation results prior to *in vivo* studies. The optimization process returned b-values of 982 ± 2 s/mm^2^ for all optimized parameter sets. Two sets (*) and (**) were examined and displayed in Table 1.

|  | Frequency of vibration [Hz] | Ratio = fv/fg | Frequency of dMEG [Hz] | Encoding efficiency [10^5^ rad/m] | min {$R(x_{c} )$} [%] | Diffusion time [ms] | Gradient amplitude [mT/m] |
| --- | --- | --- | --- | --- | --- | --- | --- |
| 1 | 25 | 0.5 | 50.0 | 2.2 | 90.9 | 50.0 | 63.6 |
| 2 | 26 | 0.5 | 52.0 | 2.2 | 89.7 | 48.1 | 67.9 |
| 3 | 27 | 0.5 | 54.0 | 2.3 | 88.3 | 46.3 | 72.4 |
| 4 | 28 | 0.5 | 56.0 | 2.3 | 86.9 | 44.6 | 77.0 |
| 5 | 29 | 0.75 | 38.7 | 2.5 | 84.5 | 47.4 | 49.2 |
| 6 | 30 | 0.75 | 40.0 | 2.5 | 82.8 | 45.8 | 52.0 |
| 7 | 31 | 0.75 | 41.3 | 2.6 | 81.0 | 44.4 | 54.8 |
| 8 | 32 | 0.75 | 42.7 | 2.6 | 79.0 | 43.0 | 57.8 |
| 9 | 33 | 0.75 | 44.0 | 2.7 | 76.9 | 41.7 | 60.8 |
| 10 | 33 | 1 | 33.0 | 2.2 | 84.1 | 45.5 | 42.5 |
| 11 | 34 | 1 | 34.0 | 2.2 | 82.6 | 44.1 | 44.6 |
| 12 | 35 | 1 | 35.0 | 2.3 | 81.1 | 42.9 | 46.7 |
| 13 | 36 | 1 | 36.0 | 2.3 | 79.4 | 41.7 | 48.9 |
| 14 | 37 | 1 | 37.0 | 2.4 | 77.6 | 40.5 | 51.1 |
| 15 | 38 | 1 | 38.0 | 2.4 | 75.7 | 39.5 | 53.4 |
| 16 | 39 | 1.25 | 31.2 | 1.6 | 88.5 | 41.7 | 42.1 |
| 17 | 40* | 1.25* | 32.0* | 1.6* | 87.6* | 40.6* | 43.8* |
| 18 | 41 | 1.25 | 32.8 | 1.6 | 86.6 | 39.6 | 45.6 |
| 19 | 42 | 1.25 | 33.6 | 1.6 | 85.6 | 38.7 | 47.4 |
| 20 | 43 | 1.25 | 34.4 | 1.7 | 84.6 | 37.8 | 49.2 |
| 21 | 44 | 1.25 | 35.2 | 1.7 | 83.4 | 36.9 | 51.1 |
| 22 | 45 | 1.25 | 36.0 | 1.7 | 82.3 | 36.1 | 53.0 |
| 23 | 46 | 1.25 | 36.8 | 1.7 | 81.0 | 35.3 | 54.9 |
| 24 | 47 | 1.25 | 37.6 | 1.8 | 79.8 | 34.6 | 56.8 |
| 25 | 48 | 1.25 | 38.4 | 1.8 | 78.5 | 33.9 | 58.8 |
| 26 | 49 | 1.25 | 39.2 | 1.8 | 77.1 | 33.2 | 60.8 |
| 27 | 50** | 1.25** | 40.0** | 1.8** | 75.7** | 32.5** | 62.8** |
| 28 | 58 | 1.25 | 46.4 | 1.5 | 76.7 | 45.3 | 61.6 |
| 29 | 59 | 1.25 | 47.2 | 1.6 | 75.4 | 44.5 | 63.4 |
